# Review of *Career Development in Bioengineering and Biotechnology *by Guruprasad Madhavan, Barbara Oakley, and Luis Kun (editors), Springer Science+Business Media, LLC, 2008

**DOI:** 10.1186/1754-1611-2-16

**Published:** 2008-11-25

**Authors:** Mark R Riley

**Affiliations:** 1 Department of Agricultural and Biosystems Engineering, The University of Arizona, Tucson, AZ 85721, USA

## Review

As an educator in biological engineering, I am often asked by students about career opportunities in this growing field. The field of biological engineering merges approaches and methodologies from a variety of traditional disciplines, and so practitioners of biological engineering can find professional opportunities in numerous areas.

The recently published book *Career Development in Bioengineering and Biotechnology*, (485 pages) edited by Madhavan, Oakley, and Kun, represents an ambitious undertaking with the goal of not only presenting career options and career development techniques but also addressing how the work of an individual can impact society. The book contains 71 chapters divided into 5 sections which cover the foundations that make biological engineers unique; traditional and alternative career paths; strategies for successful career development; and professional responsibilities. A sizeable percentage of the 85 authors, many of whom are leaders in the field, have academic training in fields quite different from biological engineering and so provide perspective from an enormous range of areas. Most importantly, contributions and perspectives are provided from Europe, Asia, and North America. A full and highly detailed index is provided. The information is practical, concrete, and would be of most use to those in the early stages of professional preparation or for more experienced personnel in search of more satisfying directions.

The approach taken here is not to describe specific companies or specific industry sectors but rather to discuss broader areas for career paths. Traditional areas include industrial research, university teaching and research, clinical engineering and research, intellectual property law, and entrepreneurship. Alternative career directions presented include regulatory affairs, energy, technology transfer, sales, and consulting. Each topic is covered with 5–10 pages of text which permits only a brief description of these job types and the steps required to reach these positions. Information on sector-based specifics (e.g. qualifications required, types of careers within these areas, day-to-day activities, and future prospects) are provided along with words of wisdom gained from each author's experience.

In Chapter 14, Rabbi Robert Shorr, Ph.D., summarizes his approach for mentoring an individual to become a successful entrepreneur in pharmaceutical and drug discovery by not only addressing internal qualities (e.g. motivation, qualifications, persistence) but also external drivers (e.g. the economy and financial requirements along with evolving technical directions). Similarly, in Chapter 7, Mark Kroll, Ph.D., describes research and management paths in industry and recommends that one focus on the 5 "P's": projects, papers, patents, presentations, and physician relationships. The first four provide validation that one's career accomplishments are new and useful. The last "P," I would extend to mean maintaining strong relationships with peers, collaborators, and other professionals in the field. These relationships are increasingly necessary in team-oriented research, development, and production environments, not to mention the increasing need for interaction with non-technically trained individuals.

A large section (52 pages) is devoted to career development and success strategies tailored toward biological engineers. Communication skills and application of career planning are major themes presented with specific examples and suggestions. One must be able to clearly articulate the nature of engineering tasks, be flexible, and be able to apply past and new knowledge to evolving professional challenges.

The last 100 or so pages provide a wealth of information on social responsibilities that reach beyond the normal professional boundaries. In addition to topics of ethics and safety, much discussion is provided on sustainability, for enhancing human welfare, and for developing appropriate collaborations. These include discussions on appropriate medical technologies for developing countries where diagnostics and therapeutics are of a lower priority than rudimentary medical tools [Lovell, pp 375].

In the chapter written by Reverend John C. Maxwell, 10 questions are provided for development of individual leadership (pp 450–452). Question number 1 is, "Am I investing in myself?" which asks about one's personal growth and whether one has reached the stage of being a life-long learner. Those who can answer in the affirmative should be developing and investing in their personal growth, applying their knowledge, and passing this information along to others. This book, when presented to practitioners of biological engineering early in their careers should satisfy all of these conditions.

One area that I would have liked to have seen covered in more depth in this text are the growing number of online databases for professional opportunities in biological engineering. For example, the Institute for Biological Engineering (IBE, the societal supporter of the *Journal of Biological Engineering*) has an online database for the posting of resumes and positions in biological engineering (IBE Career Center). Useful websites can be found peppered throughout the text, but a comprehensive and organized list in one place would be helpful. Additionally, the topics are weighted towards biomedical and biotechnological applications and could have been written with more attention paid towards environmental, bio-energy, and other newly-developing industrial sectors. These concerns are certainly minor and do not detract from this very practical and useful book.

I recommend this text for anyone interested in learning more about career options in biological engineering, how to reach these targets, or how to enhance their impact on society through professional activities.

## Competing interests

The author declares that he has no competing interests.
